# DMI fungicide resistance in *Zymoseptoria tritici* is unlinked to geographical origin and genetic background: a case study in Europe

**DOI:** 10.1002/ps.8514

**Published:** 2024-11-06

**Authors:** Eula Gems Oreiro, Berit Samils, Steven Kildea, Thies Heick, Pierre Hellin, Anne Legrève, Bernd Rodemann, Gunilla Berg, Lise N Jørgensen, Hanna Friberg, Anna Berlin, Jiasui Zhan, Björn Andersson

**Affiliations:** ^1^ Department of Forest Mycology and Plant Pathology Swedish University of Agricultural Sciences Uppsala Sweden; ^2^ TEAGASC, The Agriculture and Food Development Authority Carlow Ireland; ^3^ Department of Agroecology Aarhus University Flakkebjerg Denmark; ^4^ Plant and Forest Health Unit Walloon Agricultural Research Center Gembloux Belgium; ^5^ Applied Microbiology, Earth and Life Institute Université catholique de Louvain Louvain‐la‐Neuve Belgium; ^6^ Department of Mycology and Virology Julius Kühn‐Institut Braunschweig Germany; ^7^ Swedish Board of Agriculture Landskrona Sweden

**Keywords:** Septoria tritici blotch, population structure, fungicide resistance, *CYP51*, haplotypes

## Abstract

**BACKGROUND:**

The hemibiotrophic fungus *Zymoseptoria tritici* causing Septoria tritici blotch (STB), is a devastating foliar pathogen of wheat worldwide. A common group of fungicides used to control STB are the demethylation inhibitors (DMIs). DMI fungicides restrict fungal growth by inhibiting the sterol 14‐α‐demethylase, a protein encoded by *CYP51* gene and essential for maintaining fungal cell permeability. However, the adaptation of *Z. tritici* populations in response to intensive and prolonged DMI usage has resulted in a gradual shift towards reduced sensitivity to this group of fungicides. In this study, 311 isolates were collected pre‐treatment from nine wheat‐growing regions in Europe in 2019. These isolates were analysed by high‐throughput amplicon‐based sequencing of nine housekeeping genes and the *CYP51* gene.

**RESULTS:**

Analyses based on housekeeping genes and the *CYP51* gene revealed a lack of population structure in *Z. tritici* samples irrespective of geographical origin. Minimum spanning network (MSN) analysis showed clustering of multilocus genotypes (MLGs) based on *CYP51* haplotypes, indicating an effect of selection due to DMI fungicide use. The majority of the haplotypes identified in this study have been reported previously. The diversity and frequencies of mutations varied across regions.

**CONCLUSION:**

Using a high‐throughput amplicon‐sequencing approach, we found several mutations in the *CYP51* gene combined in different haplotypes that are likely to cause fungicide resistance. These mutations occurred irrespective of genetic background or geographical origin. Overall, these results contribute to the development of effective and sustainable risk monitoring for DMI fungicide resistance. © 2024 The Author(s). *Pest Management Science* published by John Wiley & Sons Ltd on behalf of Society of Chemical Industry.

## Graphical abstract

Minimum spanning network analysis of 311 *Zymoseptoria tritici* samples revealed clustering of multilocus genotypes grouped by *CYP51* haplotypes, indicating DMI fungicide‐driven selection, independent of geographical and genetic background in Europe.

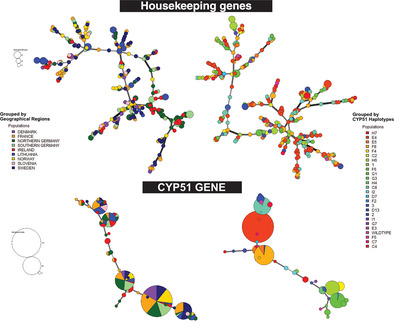

## 1 INTRODUCTION

*Zymoseptoria tritici*, a haploid hemibiotrophic fungus that causes Septoria tritici blotch (STB), is an important foliar fungal pathogen of wheat.^1^, ^2^, ^3^, ^4^ STB is distributed worldwide but is most important in wheat‐growing areas with substantial spring and summer rains.^5^ The pathogen's reproduction and spread are characterized by multiple sexual and asexual cycles throughout the growing season, which determines its epidemiology and population structure.^6^, ^7^, ^8^, ^9^, ^10^ The fruiting bodies produced by sexual reproduction contain ascospores,^5^ which can be dispersed by wind over long distances and constitute primary and secondary inoculum.^6^, ^7^, ^11^ The pathogen can also undergo asexual reproduction during which pycnidiospores are formed in pycnidia, which are locally dispersed through rain splash during the cropping season.^6^, ^11^, ^12^ *Zymoseptoria tritici* can overwinter on crop debris or living wheat plants. In infected plant and crop residues, the fungus can produce spores, both ascospores and pycnidiospores under favourable conditions, such as periods of high humidity and moderate temperatures.^13^

Yield losses due to STB can be up to 50% in untreated susceptible wheat varieties during severe epidemics.^1^, ^14^ Despite the use of resistant varieties and integrated agricultural practices, fungicide application is still the most common and effective method to minimize yield loss.^3^, ^15^ Over the past 30 years, STB control has relied heavily on demethylation inhibitors (DMI).^3^, ^15^, ^16^, ^17^, ^18^ However, *Z. tritici* has adapted in response to the intensive and prolonged use of DMI fungicides, resulting in a shift towards reduced sensitivity to many of the active ingredients in this group.^19^ DMI fungicides target the *CYP51* gene and inhibit the enzyme lanosterol 14α‐demethylase,^20^ preventing the demethylation step in the production of ergosterol, which is essential for maintaining the fluidity and permeability of the pathogen's cellular membranes. Investigating the effects of these fungicides on both the population structure and fungicide sensitivity of the pathogen under field conditions is valuable in understanding the dynamics of *Z. tritici* populations. Resistance to DMI fungicides is caused by several mechanisms including mutations in the target gene *CYP51*, overexpression of the *CYP51* gene and an increased efflux of active fungicides.^21^ Here, we focused on the most common mechanism, which is the accumulation of mutations in the *CYP51* gene leading to amino acid changes in the *CYP51* enzyme. This mechanism is considered to be the most predominant form of resistance contributing to the erosion of fungicide sensitivity of European *Z. tritici* populations in the field.^19^

The development of reduced sensitivity in *Z. tritici* has been reported in previous studies, and these results were reflected in lower field performance for control, especially for DMIs such as epoxiconazole, prothioconazole, tebuconazole and metconazole.^19^, ^22^, ^23^ Prior to 2005, DMIs such as epoxiconazole and prothioconazole did not show reduced field performance.^3^, ^18^ The later observed decline in the efficacy of DMI fungicides was primarily associated with alterations in the *CYP51* gene, where a number of mutations in the *CYP51* gene have emerged.^19^, ^21^ In addition, these mutations occur in combinations in *Z. tritici* individuals resulting in populations made up of diverse *CYP51* haplotype profiles.^17^, ^21^

Genetic diversity and population structure have been well studied in *Z. tritici*.^24^ Previous investigations on population genetic studies of *Z. tritici* using various genetic markers such as restriction fragment length polymorphism, amplified fragment length polymorphism and simple sequence repeat markers show high genetic diversity, low population differentiation and substantial gene flow between *Z. tritici* populations in the sampled geographical areas.^12^, ^24^, ^25^, ^26^, ^27^, ^28^, ^29^, ^30^ The recent development of multiplex amplification assays of genes of interest, including those involved in fungicide resistance, can be used to effectively capture the genetic variability of fungal populations.^31^, ^32^ This is important for further understanding of the biology, epidemiology and evolutionary history of *Z. tritici*.^8^, ^26^

Monitoring resistance to DMI fungicides is crucial for developing effective crop protection strategies, particularly in Europe where a decreasing frequency of mutations linked to fungicide resistance from west to east had been observed.^3^ In this study, we used a high‐throughput sequencing assay^31^ on a pan‐European collection of *Z. tritici* isolates to determine the presence of potential mutations in the *CYP51* gene associated with resistance to DMI fungicides. In addition, in the same set of isolates we sequenced nine housekeeping genes to determine whether a population structure existed in the collection.

## 2 MATERIALS AND METHODS

### 2.1 Pathogen collection, isolation and DNA extraction

**Figure 1:**
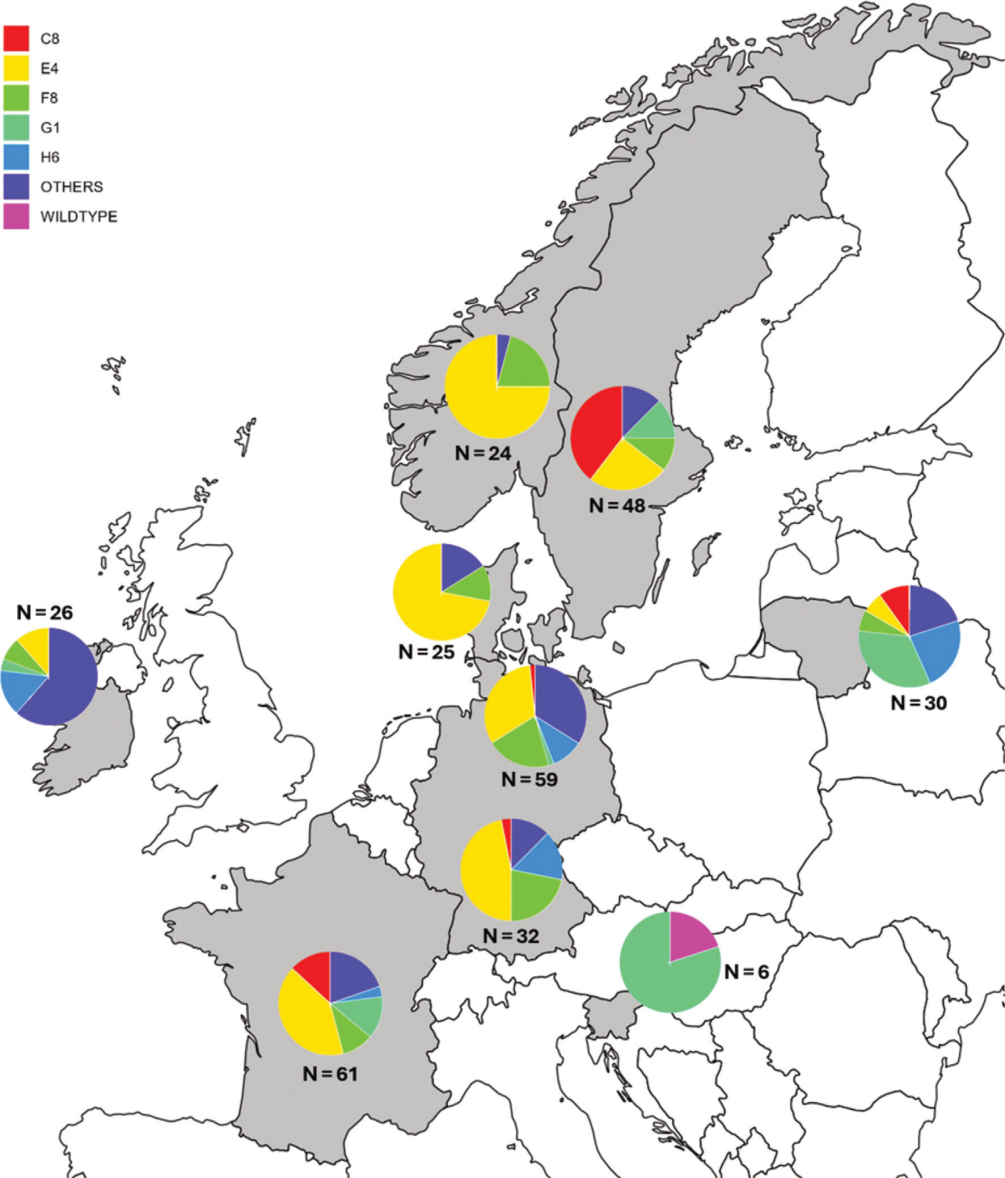
Distribution and frequency of the five most common *CYP51* haplotypes based on samples collected in 2019 from nine regions in Europe. Others represent pooled reported and unreported haplotypes with less than 7% frequency in the samples.

Leaves of winter wheat infected with STB were collected during the spring of 2019 from wheat‐growing regions in Europe, namely France, Denmark, Germany, Ireland, Lithuania, Norway, Slovenia and Sweden. Germany was divided into two regions, northern and southern Germany because of the different climatic conditions (Fig. 1, Supporting Information, Table S1). The collection of symptomatic leaves was done pre‐treatment in commercial wheat fields before growth stage (GS) 39.^3^ A single infected leaf showing STB symptoms was collected at intervals of 10–20 m along a W‐shaped path across the fields. The sampled leaves were air‐dried and kept in paper envelopes at room temperature until the isolation of pathogen.^3^ Single spore isolates were obtained following the protocol of Dooley *et al*.^33^ and stored at −80 °C in 30% glycerol (vv^−1^). Fungal DNA was extracted from 4‐day‐old single spore isolates in potato dextrose agar culture using the E.Z.N.A. SP Plant DNA Mini Kit (Omega Bio‐Tek, Doraville, GA, USA), following the manufacturers’ instructions.

### 2.2 Sequencing assay

The sequencing assay was based on the method paper by Samils *et al*.^31^ including the amplification of the nine housekeeping genes: *actin (Act)*, *beta‐tubulin‐like gene (BTUB)*, *calmodulin (Calm)*, *cyclophilin (Cyclo)*, *elongation factor 1‐alpha (EF1)*, *glyceraldehyde‐3‐phosphate dehydrogenase (GAPDH)*, *heat stress protein 80‐1 (HSP80‐1)*, *protein kinase C (PKC)* and *transcription factor class C (TFC1)*, and the fungicide resistance gene *Cytochrome P450 14α‐sterol demethylases (CYP51)* (Supporting Information, Table S2). The four steps of the assay include amplification of the target genes, PacBio long‐read sequencing, de‐multiplexing of the sequenced reads and alignment of the sequenced reads to reference sequences to identify genetic and amino acid changes.

The genes of interest were first amplified by polymerase chain reaction (PCR) using primers with universal heel sequences, allowing for addition of specific tags or indexing primers.^31^ Because of the varying amplicon sizes, two separate multiplex PCRs were used, one for long amplicons (1200–2200 bp) and one for shorter amplicons (500–1000 bp), with extension times and primer concentrations adapted to amplify all DNA fragments in acceptable amounts. In the long‐read PCR, the primers amplifying *CYP51* were included, and in the short‐read PCR, primers for the genes *Act*, *BTUB*, *Calm*, *Cyclo*, *EF1*, *GAPDH*, *HSP80‐1*, *PKC* and *TFC1* were included. A second PCR analysis was performed directly after the first in the same reaction tubes, after adding unique forward and reverse tags (2 μm) to each reaction tube. These tags allow for the identification of different samples in a single sequencing run. All amplification and cycling conditions in the first and second PCR reactions were the same as described by Samils *et al*.^31^ for both long‐read and short‐read PCR. The only difference in the cycling condition between the short‐read and long‐read PCR was the extension time, 60 s and 2 min 30 s, respectively, in all cycles in both the first and second PCR.^31^

The PCR products for each of the 96 wells within a plate were pooled in one tube and purified with AMPure XP beads (Beckman Coulter Genomics, Danvers, MA, USA) at a ratio of 1:0.6 (PCR/AMPure solution) to remove DNA fragments shorter than 300 bp. In addition, the pools where again purified using the E.Z.N.A Cycle Pure Kit (Omega Bio‐Tek). The DNA quality and amplicon sizes were analysed using an Agilent 2100 Bioanalyser (Agilent Technologies, Santa Clara, CA, USA) and the PCR products were pooled in equal volumes before PacBio sequencing. The pooled amplicons were sequenced with the PacBio Sequel platform (Pacific Biosciences, Menlo Park, CA, USA) using the single‐molecule real‐time technology and circular consensus sequencing at SciLifeLab (NGI, Uppsala, Sweden).

To sort reads based on their sequence tags and assign them to sample of origin, the data were de‐multiplexed using the in‐house developed script (‘dettag.py’) implemented in Python 2.7. Reads with a low quality or failing to match the heel or tag sequence were discarded. The script was run with the same parameters as described by Samils *et al*.^31^ Through the process, the reads were organized into new fastq files in which the heel and tag sequences were removed.

The software Geneious Prime 2021.2 was used to align sequencing reads and identify single nucleotide polymorphisms (SNPs) and mutations, using the fastq output files from the de‐multiplexing script as input. All sequencing reads were first mapped to reference genes of the nine housekeeping genes or to reference sequences of the *CYP51* gene (using one reference sequence for each exon in *CYP51*). Consensus files for each sample were thereafter aligned to each of the reference gene sequences (Supporting Information, Table S2).

### 2.3 Data analysis

A total of 311 *Z. tritici* isolates were analysed for variation in both the housekeeping genes and the *CYP51* gene. For the housekeeping genes, variable nucleotide positions in the consensus sequences were identified and SNP markers were selected at a distance of more than 20 bp from the preceding marker to reduce linkage drag.^31^ The SNP markers from the housekeeping genes were used to evaluate the population genetic structures based on selectively neutral markers. The same SNP marker selection procedure was used for the *CYP51* gene to allow direct comparison of population genetic structure based on markers from genes under different selection pressures.

To identify mutations in the *CYP51* gene, the DNA sequences were first translated into amino acid sequences. Amino acid alterations were identified based on comparison with the reference gene (wild‐type). To classify *CYP51* amino acid haplotypes, that is the combination of different *CYP51* mutations in an isolate, the nomenclature of Huf *et al*.^17^ was used. The frequency and distribution of haplotypes across the European regions were visualized by generating pie charts in Excel (Microsoft, Redmond, WA, USA).

The population genetic characteristics of the obtained SNP markers were analysed for both the data based on the housekeeping genes and the *CYP51* gene using the Poppr package v.2.9.3.^34^, ^35^ The evenness and percentage of missing values for each locus were assessed. Only samples with <3% missing SNP markers were included in the data sets. A genotype accumulation curve was performed separately for both data sets to determine the minimum number of loci required to discriminate between samples.

To analyse the genetic diversity of the housekeeping genes and the *CYP51* gene based on geographical origin, we explored the genotypic richness and diversity of the data using the Poppr package v.2.9.3 to obtain the number of multilocus genotypes (MLG) and the Shannon–Wiener index of MLG diversity based on the clone‐corrected populations.

Genetic variation within and among populations was further determined based on analysis of molecular variance (AMOVA) implemented in Poppr with clone‐corrected data for housekeeping genes. Pairwise *PhiPT* values were calculated using the software GenAlex 6.502^36^ to further analyse the population differentiation of *Z. tritici* from different regions.

Minimum spanning networks (MSN) were constructed to visualize the genetic relationships between the MLGs related to geographical origin and *CYP51* haplotypes. All MSN analyses on the SNP data sets for both housekeeping genes and the *CYP51* gene were analysed using Provesti's distance function in the Poppr package v.2.9.3. in R. Principal component analysis (PCA) was performed to identify the genetic relationship among the *Z. tritici* samples. Neighbour‐joining (NJ) trees were also constructed using the Provesti's distance function with 1000 bootstrap re‐samplings.

## 3 RESULTS

### 3.1 Frequency and distribution of *CYP51* mutations and haplotypes in *Z. tritici* samples across Europe

A total of 15 mutations were found in *Z. tritici* samples collected from nine regions in Europe. The frequency of each mutation varied between regions (Supporting Information, Fig. S1). L50S and I381V were the most common and conserved mutations present in all regions. D134G, V136A, V136C, Y461C, Y461H and S524T were also observed in all regions except Slovenia. As expected, regions with higher sample sizes had a greater diversity of *CYP51* mutations, as observed in the case of France (*n* = 61), northern Germany (*n* = 59) and Sweden (*n* = 48). In this study, we also observed rare mutations at low frequencies, Y459S (*n* = 2) in France and Y459D (*n* = 3) in Sweden (Supporting Information, Fig. S1).

Different *CYP51* haplotype distributions and frequencies were observed among the *Z. tritici* samples from different regions (Table 1, Fig. 1). Out of the 311 *Z. tritici* isolates screened for amino acid alterations in *CYP51*, only two isolates were identified as wild‐type (Table 1). A total of 25 haplotypes were detected, including three previously unreported haplotypes. Among the previously reported haplotypes,^17^ the five most common were E4, F8, C8, G1 and H6, representing 36.0%, 13.5%, 10.3%, 9.7% and 7.72% of the samples, respectively (Table 1, Fig. 1). These were followed by haplotypes D13, E5 and F4 with 2.89% each. Haplotype H7 represented 2.25% of the samples followed by F6 with 1.61%, and H4 and I2 with 1.29% each. Haplotypes C2, C4, C7, D7, E3, F2, F5, G3, G7 and I1 were found at very low frequencies ranging from 0.32% to 0.96% (Table 1). The previously reported haplotypes were mainly found in samples from northern Germany, Lithuania, Ireland and Sweden (Table 1, Fig. 1).

**Table 1:** *CYP51* haplotypes detected in 311 *Zymoseptoria tritici* isolates collected from nine regions in 2019

Haplotype	*n*	Frequency (%)	Position of amino acid alteration											Region
			L50S	D134G	V136A/C	S188N	A379G	I381V	Y459S/D/DEL	G460DEL	Y461H/S	N513K	S524T	
*Cyp51* wild‐type	NA	NA	L	D	V	S	A	I	Y	G	Y	N	S	—
0	2	0.64	—	—	—	—	—	—	—	—	—	—	—	Sl
1	2	0.64	—	—	—	—	—	V	—	—	—	K	T	GE‐N,FR
2	4	1.29	S	G	A	—	G	V	DEL	DEL	S	—	T	GE‐N,IR,LI
3	3	0.96	S	G	A	N	G	V	DEL	DEL	—	—	T	GE‐N,LI
C2	2	0.64	S	—	—	—	—	V	S	—	—	—	—	FR
C4	3	0.96	S	—	—	—	—	V	D	—	—	—	—	SW
C7	1	0.32	S	—	A	—	—	—	—	—	H	—	—	SW
C8	32	10.29	S	—	—	—	—	V	—	—	H	—	—	GE‐N,GE‐S,FR,LI,SW
D7	1	0.32	S	—	A	—	—	—	—	—	S	—	T	FR
D13	9	2.89	—	—	C	—	—	V	—	—	H	—	T	GE‐N,LI,NO
E3	1	0.32	S	—	A	—	—	V	—	—	S	—	T	IR
E4	112	36.01	S	G	A	—	—	V	—	—	H	—	—	GE‐N,GE‐S,IR,DK,FR,LI,NO,SW
E5	9	2.89	S	—	A	—	—	V	—	—	H	—	T	GE‐S,IR,DK,GE‐N,SW
F2	1	0.32	S	—	—	N	—	V	DEL	DEL	—	K	—	FR
F4	9	2.89	S	—	C	N	—	V	—	—	H	—	T	GE‐N,GE‐S,IR,DK
F5	1	0.32	S	—	A	N	—	—	DEL	DEL	—	K	—	SW
F6	5	1.61	S	—	A	N	—	—	DEL	DEL	—	—	T	GE‐N,IR,FR
F8	42	13.5	S	G	A	—	—	V	—	—	H	—	T	GE‐N,GE‐S,IR,DK,FR,LI,NO,SW
G1	30	9.65	S	—	—	N	G	V	DEL	DEL	—	K	—	GE‐N,IR,FR,LI,SL,SW
G3	1	0.32	S	—	A	N	—	—	DEL	DEL	—	K	T	FR
G7	1	0.32	S	—	A	N	G	V	—	—	S	—	T	IR
H4	4	1.29	S	—	A	N	G	V	DEL	DEL	—	—	T	GE‐N,IR,FR
H6	24	7.72	S	—	C	N	G	V	DEL	DEL	—	—	T	GE‐N,GE‐S,IR,FR,LI
H7	7	2.25	S	G	A	—	G	V	DEL	DEL	—	—	T	GE‐N,IR,DK,FR
I1	1	0.32	S	—	A	N	G	V	DEL	DEL	—	K	T	IR
I2	4	1.29	S	G	A	—	G	V	DEL	DEL	—	K	T	GE‐N,IR,FR

Haplotype 0 corresponds to *CYP51* wild‐type. DEL, deletion of the amino acid; *n*, total number of isolates detected in each haplotype; NA, not applicable. DK, Denmark; FR, France; GE‐N, northern Germany; GE‐S, southern Germany; IR, Ireland; LI, Lithuania; NO, Norway; SL, Slovenia; SW, Sweden.

The unreported *CYP51* haplotypes representing 2.9% of the total samples in this study were designated as haplotype 1, haplotype 2 and haplotype 3, representing 0.64% to 1.29% of the samples (Table 1). These unreported haplotypes were identified in samples from France, northern Germany, Ireland and Lithuania.

### 3.2 Genetic diversity

We used 113 SNP loci from nine housekeeping genes in the 311 *Z. tritici* samples (Supporting Information, Table S3), the same set of SNP loci identified by Samils *et al*.^31^ The number of identified alleles in each locus varied from one to three. The values of evenness ranged between 0.33 for the locus ACT115 and 1.00 for the locus TFC1‐336, indicating that the alleles were unevenly distributed in the samples. The overall percentage of missing values was low, ranging from 0.29% to 3.42% between the loci. The genotype accumulation curve reached saturation, indicating that the number of loci in housekeeping genes used in this study was sufficient to discriminate genotypic variation in the data (Supporting Information, Fig. S2). For the *CYP51* gene, a total of 21 SNP loci were selected. All loci had two alleles, except locus 1_407, which had three alleles. The values of evenness varied between 0.33 for 1_320 to 0.98 for 4_738 and 4_768. There were no missing values among the loci (Supporting Information, Table S4). The genotype accumulation curve for the *CYP51* gene, which is under selection, did not reach saturation (Supporting Information, Fig. S3).

For the housekeeping genes, a total of 274 MLGs were identified across the nine European regions based on 113 SNPs, as shown in Table 2. The number of MLGs in the populations from France, Slovenia and Sweden was equal to the number of samples from each of these regions. No MLGs were shared between the regions. The Shannon–Wiener index (*H*) values ranged from 1.79 to 4.11, with the highest value observed in the population from France. These indices signify genotypic richness, with higher values indicating a more diverse array of MLGs in the populations studied. For the *CYP51* gene, a total of 34 MLGs were identified, 14 MLGs were shared between most regions, whereas 20 were identified as unique to a particular region (Supporting Information, Table S1). The *H* values ranged from 0.69 to 2.83 (Table 2).

**Table 2:** Population genetic diversity in *Zymoseptoria tritici* populations based on single nucleotide polymorphism markers in the housekeeping genes and the *CYP51* gene

Regions	*N*	Housekeeping genes		*CYP51*	
		MLG	H	MLG	H
Denmark	25	23	3.14	4	1.39
France	61	61	4.11	14	2.64
Northern Germany	59	51	3.93	17	2.83
Southern Germany	32	24	3.18	6	1.79
Ireland	26	24	3.18	13	2.57
Lithuania	30	14	2.64	8	2.08
Norway	24	23	3.14	3	1.10
Slovenia	6	6	1.79	2	0.69
Sweden	48	48	3.87	11	2.40
All regions	311	274	5.61	34	3.23

*N*, number of samples; MLG, multilocus genotypes; H, Shannon–Wiener index of MLG.

### 3.3 Comparison between population structure based on housekeeping genes *versus* *CYP51* fungicide resistance gene

**Figure 2:**
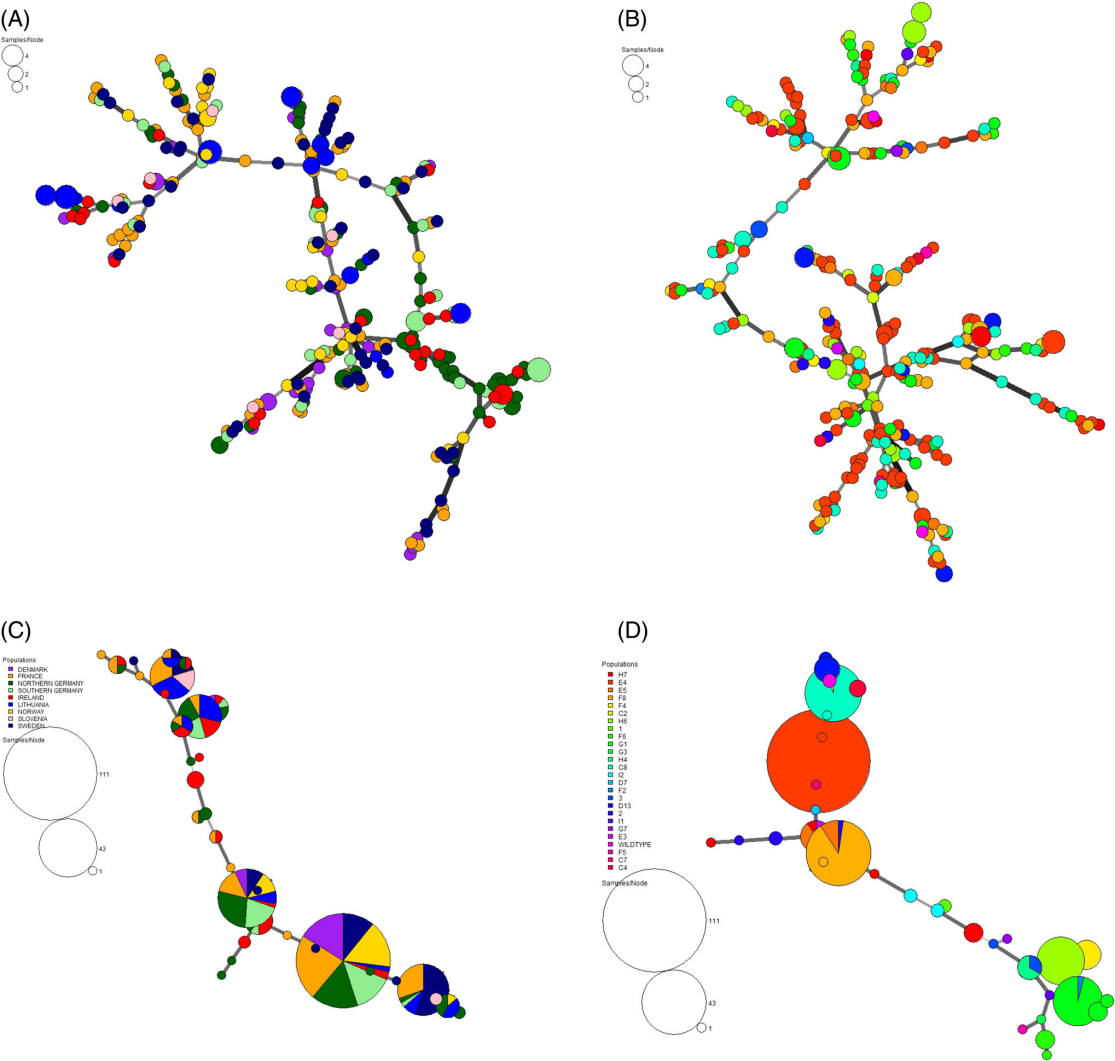
Minimum spanning network (MSN) of *Zymoseptoria tritici* based on Provesti's distance. Each circle represents a unique multilocus genotype (MLG), branch thickness represents genetic relatedness and MLGs shared between regions or haplotypes are indicated by split nodes. MSN based on (a) single nucleotide polymorphism (SNP) markers in housekeeping genes, colour indicates region. (b) SNP markers in housekeeping genes, colour indicates *CYP51* haplotype (Table 1). (c) SNP markers in *CYP51* gene, colour indicates region. (d) SNP markers in *CYP51* gene, colour indicates *CYP51* haplotype.

MSN analyses of the housekeeping genes showed no clear associations of MLGs with geographical origin and haplotypes (Fig. 2(a),(b)). The PCA and NJ trees showed similar results (data not shown). By contrast, the results of the AMOVA showed that 91.96% (*P* = 0.001) of the total variance was found within samples, whereas 5.71% (*P* = 0.001) was due to variation between regions (Table 3). Individual MSN analysis was also performed for each of the housekeeping genes. The differences observed between the MSNs correlate with the number of SNPs available in each housekeeping gene, where more SNPs resulted in less‐structured MSN (Supporting Information, Fig. S4).

**Table 3:** Analysis of molecular variance in *Zymoseptoria tritici* populations using 113 single nucleotide polymorphism markers

Source	df	SS	MS	Variance components	Variation (%)	*P*‐value
Variation between regions	8	700.66	87.58	1.52	5.71	0.001
Variation between samples within regions	15	454.91	30.33	0.62	2.34	0.022
Variation within samples	287	7049.14	24.56	24.56	91.96	0.001
Total	310	8204.71	26.47	26.71	100.00	—

DF, degrees of freedom; SS, sum of squares; MS, mean sum of squares.

Pairwise comparisons of populations from the different regions showed significant differentiation between some regions, whereas others had low and non‐significant differentiation (Table 4). The highest and significant values of pairwise *PhiPT* were found between populations of southern Germany and all other regions except Denmark and Slovenia. In comparison, Slovenia and southern Germany, Slovenia and northern Germany, Slovenia and Denmark, Norway and France, southern Germany and Denmark, and Sweden and Lithuania did not show any significant differentiation (Table 4).

**Table 4:** Pairwise *PhiPT* values between the *Zymoseptoria tritici* populations using 113 single nucleotide polymorphism markers

	Ireland	France	Slovenia	Southern Germany	Northern Germany	Denmark	Norway	Sweden	Lithuania
Ireland	—	—	—	***	*	**	*	**	—
France	0.013	—	—	***	**	**	—	—	—
Slovenia	0.022	0.025	—	—	—	—	—	—	—
Southern Germany	**0.106**	**0.105**	0.000	—	*	—	***	***	***
Northern Germany	**0.032**	**0.033**	0.000	**0.033**	—	—	**	**	*
Denmark	**0.053**	**0.043**	0.000	0.000	0.000	—	**	**	*
Norway	**0.050**	0.000	0.058	**0.140**	**0.050**	**0.056**	—	—	—
Sweden	**0.030**	0.005	0.029	**0.114**	**0.041**	**0.053**	0.023	—	—
Lithuania	0.031	0.016	0.046	**0.128**	**0.042**	**0.060**	0.037	0.000	—

*PhiPT* values are shown below diagonal. Significant *p‐*values <0.05 are shown in bold *PhiPT* values. Probability, P(rand ≥ data) based on 999 permutations is shown above the diagonal. *indicates *p* <0.05, **indicates *p* <0.01, *** indicates *p* <0.001.

The MSN based on SNP markers in the *CYP51* gene showed clustering of MLGs independent of geographical origin (Fig. 2(c)). This was also shown in the phylogenetic tree using the NJ tree approach (data not shown). The smallest nodes along the branch represent unique MLGs, most of which belonged to samples collected in France, northern Germany, Ireland and Sweden (Fig. 2(c), Table S1). MSN analysis was also performed to visualize the relationships between MLGs and the *CYP51* haplotypes. This analysis revealed, as expected, a clear grouping of *Z. tritici* samples with the same haplotypes distributed in most cases across the MLGs. There were also MLGs shared between more genetically similar haplotypes as shown by five split nodes (Fig. 2(d)). Based on the *CYP51* MSN indicated by haplotypes, the distribution of the reported and unreported haplotypes revealed no pattern of clustering in the network. The largest node in the *CYP51* MSN grouped by haplotypes (Fig. 2(d)) correlates with the haplotype E4, the one with the highest frequency among all samples (Supporting Information, Table S1).

## 4 DISCUSSION

The use of DMI fungicides to manage STB has the drawback of selecting for resistant strains, which will reduce the efficacy of specific active ingredients. Thus, it is important to monitor the distribution of *CYP51* mutations in *Z. tritici* populations to design effective disease management strategies. In this study, we used a high‐throughput sequencing assay to determine the population structure and *CYP51* haplotypes potentially associated with fungicide sensitivity in European *Z. tritici* samples. This sequencing method, based on PacBio long‐read sequencing technology,^31^ is more efficient than other molecular assays used to screen for fungicide sensitivity. It can sequence multiple amplicons in a single run and capture all alleles present in a gene and, as a result, speed up the process of genotyping fungicide resistance.

Geographical separation contributes to the differentiation of pathogen populations.^37^, ^38^ Even though we based our study on a pan‐European sample collection, the MSN analyses of the nine housekeeping genes showed a lack of structure and clusters related to geographical origin (Fig. 2(a)). The presence of unique MLGs was scattered throughout the network, as shown in Fig. 2(a), and the weak structure of *Z. tritici* populations between regions supported by the low and non‐significant pairwise *PhiPT* values (Table 4), suggests high gene flow in the wheat–*Z. tritici* pathosystem when genes not under selection are used to infer the population structure. The pathogen *Z. tritici* is capable of long‐distance dispersal, mainly by wind‐dispersed ascospores, and also by wind‐dispersed clonal pycnidiospores, but only over shorter distances. This will cause successive infection events and drive the progression of STB in wheat‐growing areas and leading to high gene flow between populations. This result was expected and agrees with previous reports of high genotypic diversity of *Z. tritici* in Europe^25^, ^26^, ^27^, ^28^, ^29^, ^30^ explained by the sexual recombination within the mixed reproduction mode of the pathogen leading to a high generation rate of new genotypes.^8^, ^12^ In this study, we used a combination of nine housekeeping genes to improve the resolution of genetic structure analyses, although a single housekeeping gene could be used if the number of SNPs is sufficient to discriminate between the included samples.

We compared the genetic diversity of the *Z. tritici* samples based on housekeeping genes with a gene under selection, the *CYP51* gene. The MSN analysis based on SNP markers of the *CYP51* gene showed no clear structure attributed to the geographical origin of the *Z. tritici* samples. As expected, the MLGs based on SNP markers grouped by *CYP51* haplotypes showed a clear clustering, because the majority of the individuals with the same haplotypes were clustered together in the same nodes. MLG32 as an example was the largest node and was linked to haplotype E4. We also found MLGs (MLG2, MLG11, MLG18, MLG33 and MLG34) shared between haplotypes (Fig. 2(d), Supporting Information, Table S1); MLG33, for example, included the haplotypes D13, E5 and F8. The differences in MLG clustering between the SNP markers of the *CYP51* gene based on geographical origin (Fig. 2(c)) and *CYP51* haplotypes (Fig. 2(d)) can be explained by the selection of SNP variability when processing the *CYP51* genetic data sets for MSN analyses. We considered at least 20 bp distance between SNPs on the *CYP51* DNA gene sequence to reduce linkage drag^31^ in the selection of SNPs.

The accumulation of mutations in the *CYP51* gene is complex because combinations of these mutations generate haplotypes that show different levels of fungicide resistance in the field.^17^, ^21^, ^39^ Therefore, an analysis that allows complete haplotype identification is important in field monitoring to describe both current and potential levels of fungicide resistance in *Z. tritici* populations. In this study, five of the most frequently observed haplotypes (E4, F8, C8, G1 and H6) align to previously reported studies of *CYP51* haplotypes.^17^, ^21^ Haplotype E4, which is the largest node in the network is likely to influence the population structure of *Z. tritici* because of selection posed by fungicide applications. Among the haplotypes, it appears that E4 has a strong effect on the level of fungicide resistance. It was found in 36% of the total samples (Fig. 1 and Table 1) indicating that the combinations of mutations present in haplotype E4 (Table 1) give a selective advantage that is also reflected by the widespread distribution of E4 in different regions (Fig. 1).

The high number of haplotypes reported in Denmark, northern and southern Germany, France, Ireland, Lithuania, Norway and Sweden indicate the high adaptation potential in the pathogen (Table 1).^40^ Few mutations were observed in the population from Slovenia (Supporting Information, Fig. S1), which may be related to the small sample size, thus variations and adaptation of the isolates could not be fully verified. In addition, the variation in the frequencies of unreported haplotypes (haplotype 1, haplotype 2 and haplotype 3) and reported haplotypes with low frequencies (<7%) in the samples (Table 1), which are labelled ‘others’ in Fig. 1, is likely due to a variable profile of *CYP51* mutations that have emerged and been selected by local fungicide use or by random dispersal of ascospores.^19^, ^41^

The heterogenous frequencies and distribution of *CYP51* haplotypes across the European regions may also be due to specific DMIs.^18^ DMI fungicides with different active ingredients are associated with specific *CYP51* mutations. For example, the highly prevalent haplotype E4 carries five mutations (L50S, D134G, V136A, I381V and Y461H), each of which and the combined effect of the interacting mutations may have a different response to each of the DMI active ingredient. In addition, exposure of *Z. tritici* to different application strategies, e.g. number of treatments, application rates and use of azoles alone or in mixture vary by season and region in Europe and will also influence the selection of *CYP51* mutations.^18^, ^42^ Here, we also checked the distribution of individual mutations composing the *CYP51* haplotypes, because information of new and arising mutations may be important in explaining the trend in DMI fungicide resistance across the regions. The S524T mutation is of interest because this mutation is correlated with decreased sensitivity to epoxiconazole and prothioconazole, as demonstrated in a previous study.^43^ In another study, the S524T mutation was also observed to be associated with fungicide resistance and its frequency gradually decreased from west to east in Europe.^3^, ^19^ Our data showed that 41% of the haplotypes detected in our study were in combination with S524T. Although among the common haplotypes presented, the S524T mutation was found only in F8 and H6. Nevertheless, the occurrence and increase in this additional amino acid alteration is worth monitoring because it has been previously reported to have a significant impact on sensitivity to demethylation inhibitors.^3^, ^43^

The use of DMI varies across Europe because all countries have different fungicide recommendations and legislation, leading to differences in fungicide use that may have an impact on the selection of *CYP51*.^18^ Further, because the collection we used in this study was sampled before fungicide applications, the identified *CYP51* mutations have most likely been present during previous growing seasons or were the result of migration. In addition, even though we do not have phenotype data for our samples, our MSN results highlight the association between specific haplotypes and selection caused by fungicide resistance.

Overall, the identification of haplotypes is relevant for resistance monitoring because *CYP51* remains under continuous selection pressure from the intensive use of DMI fungicides.^17^, ^19^ To facilitate a more meaningful comparison of haplotypes, better information on disease pressure from samples sources, fungicide use combined with a more structured sampling strategy and fungicide sensitivity testing would be valuable. The PacBio long‐read sequencing assay, despite its higher initial setup costs, is an efficient amplicon‐sequencing assay that can be applied to larger collections of *Z. tritici* to provide a comprehensive overview of the genetic monitoring of DMI fungicide resistance in wheat‐growing regions in Europe.

## 5 CONCLUSION

This study used a high‐throughput sequencing method capable of analysing long‐read sequences to detect mutations in the entire *CYP51* gene. Fungicide selection influences the population structure of *Z. tritici* samples regardless of genetic background and geographical origin. The approach used in this study is a promising diagnostic tool to identify known and potential combinations of mutations that may be associated with DMI fungicide resistance. The results presented provide valuable insights for effective and sustainable monitoring of the prevalence and risk of DMI fungicide resistance within the *Z. tritici* population in Europe.

## CONFLICT OF INTEREST

The authors declare that they have no conflict of interest.

## Supporting information


**Data S1.** Supporting information.

## Data Availability

The data that supports the findings of this study are available in the supplementary material of this article.
