# Supplementary material for: DMI fungicide resistance in Zymoseptoria tritici is unlinked to geographical origin and genetic background: a case study in Europe
Source: Pest Manag Sci. 2024 Nov 6;81(2):1103–12. doi: 10.1002/ps.8514 (PMC11716363; doi:10.1002/ps.8514)
Supplement: Supplementary file 1 — Data S1. Supporting information. [file PS-81-1103-s001.docx]

**Supplementary Information**

Table S1. Identity of 311 *Zymoseptoria tritici* isolates collected in 2019 that were included in this study.

|  | **Sample ID** | **Country** | **Region** | **Subregion** | **Location** | **MLG ID Number for Housekeeping Genes** | **MLG ID Number for CYP51 Gene** | **CYP51 Haplotype** |
| --- | --- | --- | --- | --- | --- | --- | --- | --- |
| 1 | DK77 | Denmark | Denmark | Sealand | Flakkebjerg | 130 | 33 | E5 |
| 2 | DK78 | Denmark | Denmark | Sealand | Flakkebjerg | 135 | 32 | E4 |
| 3 | DK79 | Denmark | Denmark | Sealand | Flakkebjerg | 129 | 34 | H7 |
| 4 | DK82 | Denmark | Denmark | Sealand | Flakkebjerg | 63 | 32 | E4 |
| 5 | DK85 | Denmark | Denmark | Sealand | Flakkebjerg | 70 | 33 | F8 |
| 6 | DK86 | Denmark | Denmark | Sealand | Flakkebjerg | 236 | 32 | E4 |
| 7 | DK87 | Denmark | Denmark | Sealand | Flakkebjerg | 105 | 32 | E4 |
| 8 | DK88 | Denmark | Denmark | Sealand | Flakkebjerg | 63 | 32 | E4 |
| 9 | DK89 | Denmark | Denmark | Sealand | Flakkebjerg | 252 | 32 | E4 |
| 10 | DK90 | Denmark | Denmark | Sealand | Flakkebjerg | 41 | 33 | F8 |
| 11 | DK92 | Denmark | Denmark | Sealand | Flakkebjerg | 56 | 32 | E4 |
| 12 | DK93 | Denmark | Denmark | Jutland | Horsens | 261 | 32 | E4 |
| 13 | DK94 | Denmark | Denmark | Jutland | Horsens | 213 | 32 | E4 |
| 14 | DK95 | Denmark | Denmark | Jutland | Horsens | 95 | 32 | E4 |
| 15 | DK96 | Denmark | Denmark | Jutland | Horsens | 81 | 32 | E4 |
| 16 | DK97 | Denmark | Denmark | Jutland | Horsens | 205 | 32 | E4 |
| 17 | DK98 | Denmark | Denmark | Jutland | Horsens | 212 | 32 | E4 |
| 18 | DK99 | Denmark | Denmark | Jutland | Horsens | 120 | 32 | E4 |
| 19 | DK101 | Denmark | Denmark | Jutland | Horsens | 141 | 32 | E4 |
| 20 | DK102 | Denmark | Denmark | Jutland | Horsens | 69 | 32 | E4 |
| 21 | DK103 | Denmark | Denmark | Jutland | Horsens | 177 | 32 | E4 |
| 22 | DK104 | Denmark | Denmark | Jutland | Horsens | 17 | 13 | F4 |
| 23 | DK105 | Denmark | Denmark | Jutland | Horsens | 30 | 34 | F8 |
| 24 | DK108 | Denmark | Denmark | Jutland | Horsens | 67 | 34 | E5 |
| 25 | DK110 | Denmark | Denmark | Jutland | Horsens | 177 | 32 | E4 |
| 26 | FR3 | France | France | Bretagne | Bignan | 264 | 20 | C2 |
| 27 | FR5 | France | France | Bretagne | Bignan | 113 | 32 | E4 |
| 28 | FR6 | France | France | Bretagne | Bignan | 47 | 20 | C2 |
| 29 | FR8 | France | France | Bretagne | Bignan | 260 | 33 | F8 |
| 30 | FR9 | France | France | Bretagne | Bignan | 2 | 32 | E4 |
| 31 | FR10 | France | France | Bretagne | Bignan | 262 | 32 | E4 |
| 32 | FR11 | France | France | Bretagne | Bignan | 40 | 5 | 1 |
| 33 | FR13 | France | France | Bretagne | Bignan | 176 | 33 | F8 |
| 34 | FR14 | France | France | Bretagne | Bignan | 263 | 12 | H6 |
| 35 | FR16 | France | France | Bretagne | Bignan | 195 | 32 | E4 |
| 36 | FR17 | France | France | Bretagne | Bignan | 206 | 32 | E4 |
| 37 | FR18 | France | France | Bretagne | Bignan | 165 | 28 | H7 |
| 38 | FR19 | France | France | Bretagne | Bignan | 186 | 32 | E4 |
| 39 | FR20 | France | France | Bretagne | Bignan | 207 | 11 | G1 |
| 40 | FR21 | France | France | Bretagne | Bignan | 204 | 32 | E4 |
| 41 | FR22 | France | France | Bretagne | Bignan | 33 | 16 | F6 |
| 42 | FR23 | France | France | Hauts | Aubigny | 185 | 32 | E4 |
| 43 | FR24 | France | France | Hauts | Aubigny | 121 | 15 | F2 |
| 44 | FR25 | France | France | Hauts | Aubigny | 118 | 33 | F8 |
| 45 | FR26 | France | France | Hauts | Aubigny | 251 | 32 | E4 |
| 46 | FR27 | France | France | Hauts | Aubigny | 193 | 33 | F8 |
| 47 | FR28 | France | France | Hauts | Aubigny | 97 | 32 | E4 |
| 48 | FR29 | France | France | Hauts | Aubigny | 128 | 12 | H6 |
| 49 | FR30 | France | France | Hauts | Aubigny | 184 | 32 | E4 |
| 50 | FR32 | France | France | Hauts | Aubigny | 109 | 32 | E4 |
| 51 | FR33 | France | France | Hauts | Aubigny | 82 | 32 | E4 |
| 52 | FR34 | France | France | Hauts | Aubigny | 174 | 32 | E4 |
| 53 | FR35 | France | France | Hauts | Aubigny | 66 | 27 | I2 |
| 54 | FR36 | France | France | Hauts | Aubigny | 210 | 33 | F8 |
| 55 | FR37 | France | France | Hauts | Aubigny | 124 | 32 | E4 |
| 56 | FR38 | France | France | Hauts | Aubigny | 90 | 20 | C8 |
| 57 | FR39 | France | France | Hauts | Aubigny | 111 | 18 | H4 |
| 58 | FR40 | France | France | Hauts | Aubigny | 5 | 1 | F6 |
| 59 | FR41 | France | France | Hauts | Aubigny | 235 | 33 | F8 |
| 60 | FR42 | France | France | Hauts | Aubigny | 238 | 32 | E4 |
| 61 | FR43 | France | France | Hauts | Aubigny | 194 | 32 | E4 |
| 62 | FR44 | France | France | Hauts | Aubigny | 93 | 32 | E4 |
| 63 | FR45 | France | France | Occitany | Auch | 53 | 16 | F6 |
| 64 | FR46 | France | France | Occitany | Auch | 84 | 11 | G1 |
| 65 | FR47 | France | France | Occitany | Auch | 259 | 11 | G1 |
| 66 | FR48 | France | France | Occitany | Auch | 1 | 32 | E4 |
| 67 | FR49 | France | France | Occitany | Auch | 50 | 11 | G1 |
| 68 | FR64 | France | France | Occitany | Auch | 88 | 11 | G1 |
| 69 | FR65 | France | France | GrandEst | Vraux | 11 | 20 | C8 |
| 70 | FR66 | France | France | GrandEst | Vraux | 168 | 11 | G1 |
| 71 | FR67 | France | France | GrandEst | Vraux | 102 | 32 | E4 |
| 72 | FR68 | France | France | GrandEst | Vraux | 80 | 11 | G1 |
| 73 | FR69 | France | France | GrandEst | Vraux | 112 | 32 | E4 |
| 74 | FR71 | France | France | GrandEst | Vraux | 196 | 31 | D7 |
| 75 | FR72 | France | France | GrandEst | Vraux | 104 | 20 | C8 |
| 76 | FR73 | France | France | GrandEst | Vraux | 266 | 20 | C8 |
| 77 | FR74 | France | France | GrandEst | Vraux | 230 | 32 | E4 |
| 78 | FR75 | France | France | GrandEst | Vraux | 18 | 32 | E4 |
| 79 | FR76 | France | France | GrandEst | Vraux | 255 | 9 | G1 |
| 80 | FR77 | France | France | GrandEst | Vraux | 108 | 32 | E4 |
| 81 | FR78 | France | France | GrandEst | Vraux | 167 | 20 | C8 |
| 82 | FR80 | France | France | GrandEst | Vraux | 71 | 20 | C8 |
| 83 | FR81 | France | France | GrandEst | Vraux | 246 | 32 | E4 |
| 84 | FR82 | France | France | GrandEst | Vraux | 15 | 11 | F2 |
| 85 | FR83 | France | France | GrandEst | Vraux | 202 | 20 | C8 |
| 86 | FR84 | France | France | GrandEst | Vraux | 96 | 20 | C8 |
| 87 | GE1S | Germany | Southern Germany | Bavaria | Wassertrudingen | 85 | 33 | E5 |
| 88 | GE3_319cN | Germany | Northern Germany | SchHols | Reubenkoge | 107 | 32 | E4 |
| 89 | GE4S | Germany | Southern Germany | Bavaria | Wassertrudingen | 175 | 32 | E4 |
| 90 | GE6S | Germany | Southern Germany | Bavaria | Wassertrudingen | 12 | 34 | E5 |
| 91 | GE7S | Germany | Southern Germany | Bavaria | Wassertrudingen | 254 | 32 | E4 |
| 92 | GE8_270eN | Germany | Northern Germany | SchHols | Hillgroven | 101 | 7 | D13 |
| 93 | GE9S | Germany | Southern Germany | Bavaria | Wassertrudingen | 265 | 33 | F8 |
| 94 | GE10S | Germany | Southern Germany | Bavaria | Wassertrudingen | 123 | 12 | H6 |
| 95 | GE11S | Germany | Southern Germany | Bavaria | Wassertrudingen | 32 | 32 | E4 |
| 96 | GE12S | Germany | Southern Germany | Bavaria | Wassertrudingen | 32 | 32 | E4 |
| 97 | GE13_322aN | Germany | Northern Germany | SchHols | Reubenkoge | 268 | 32 | E4 |
| 98 | GE13S | Germany | Southern Germany | Bavaria | Wassertrudingen | 219 | 20 | C8 |
| 99 | GE14S | Germany | Southern Germany | Bavaria | Wassertrudingen | 187 | 13 | F4 |
| 100 | GE15S | Germany | Southern Germany | Bavaria | Wassertrudingen | 86 | 33 | F8 |
| 101 | GE16S | Germany | Southern Germany | Bavaria | Wassertrudingen | 191 | 12 | H6 |
| 102 | GE17S | Germany | Southern Germany | Bavaria | Wassertrudingen | 127 | 32 | E4 |
| 103 | GE18S | Germany | Southern Germany | Bavaria | Wassertrudingen | 22 | 33 | F8 |
| 104 | GE19S | Germany | Southern Germany | Bavaria | Wassertrudingen | 173 | 32 | E4 |
| 105 | GE20S | Germany | Southern Germany | Bavaria | Wassertrudingen | 127 | 32 | E4 |
| 106 | GE21S | Germany | Southern Germany | Bavaria | Wassertrudingen | 183 | 32 | E4 |
| 107 | GE22S | Germany | Southern Germany | Bavaria | Wassertrudingen | 20 | 32 | E4 |
| 108 | GE24N | Germany | Northern Germany | Schl | Kastorf | 140 | 23 | I2 |
| 109 | GE28N | Germany | Northern Germany | Schl | Kastorf | 62 | 13 | F4 |
| 110 | GE29_322eN | Germany | Northern Germany | SchHols | Reubenkoge | 268 | 32 | E4 |
| 111 | GE29N | Germany | Northern Germany | Schl | Kastorf | 192 | 12 | H6 |
| 112 | GE31N | Germany | Northern Germany | Schl | Kastorf | 61 | 33 | F8 |
| 113 | GE36N | Germany | Northern Germany | Schl | Kastorf | 72 | 33 | E5 |
| 114 | GE39N | Germany | Northern Germany | Schl | Kastorf | 25 | 12 | H6 |
| 115 | GE40N | Germany | Northern Germany | Schl | Kastorf | 250 | 8 | D13 |
| 116 | GE41N | Germany | Northern Germany | Schl | Kastorf | 224 | 32 | E4 |
| 117 | GE50N | Germany | Northern Germany | LSaxony | Bunde | 197 | 13 | F4 |
| 118 | GE82N | Germany | Northern Germany | LSaxony | Einbeck | 23 | 13 | F4 |
| 119 | GE84N | Germany | Northern Germany | LSaxony | Einbeck | 203 | 33 | F8 |
| 120 | GE86N | Germany | Northern Germany | LSaxony | Einbeck | 99 | 32 | E4 |
| 121 | GE201N | Germany | Northern Germany | Niedersachsen | Friedland | 180 | 32 | E4 |
| 122 | GE203N | Germany | Northern Germany | Niedersachsen | Friedland | 220 | 33 | F8 |
| 123 | GE204N | Germany | Northern Germany | Niedersachsen | Friedland | 180 | 3 | E4 |
| 124 | GE208N | Germany | Northern Germany | Niedersachsen | Friedland | 233 | 34 | F8 |
| 125 | GE213N | Germany | Northern Germany | LSaxony | Einbeck | 117 | 32 | E4 |
| 126 | GE219N | Germany | Northern Germany | LSaxony | Einbeck | 242 | 10 | G1 |
| 127 | GE223N | Germany | Northern Germany | Niedersachsen | EinbeckHolt | 65 | 32 | E4 |
| 128 | GE227N | Germany | Northern Germany | Niedersachsen | EinbeckHolt | 98 | 32 | E4 |
| 129 | GE228N | Germany | Northern Germany | Niedersachsen | EinbeckHolt | 98 | 32 | E4 |
| 130 | GE235N | France | France | Bretagne | Bignan | 126 | 32 | E4 |
| 131 | GE237N | Germany | Northern Germany | SachsA | Halle | 60 | 13 | F4 |
| 132 | GE239N | Germany | Northern Germany | SachsA | Halle | 126 | 32 | E4 |
| 133 | GE241N | Germany | Northern Germany | LSaxony | Bunde | 28 | 33 | F8 |
| 134 | GE243N | Germany | Northern Germany | LSaxony | Bunde | 92 | 13 | F4 |
| 135 | GE245N | Germany | Northern Germany | LSaxony | Bunde | 94 | 33 | F8 |
| 136 | GE247N | Germany | Northern Germany | LSaxony | Bunde | 237 | 32 | E4 |
| 137 | GE252N | Germany | Northern Germany | MeckV | Karstorf | 234 | 21 | 3 |
| 138 | GE253N | Germany | Northern Germany | MeckV | Karstorf | 223 | 12 | H6 |
| 139 | GE254N | Germany | Northern Germany | MeckV | Karstorf | 190 | 25 | H7 |
| 140 | GE256N | Germany | Northern Germany | MeckV | Karstorf | 229 | 33 | F8 |
| 141 | GE257N | Germany | Northern Germany | MeckV | Karstorf | 229 | 33 | F8 |
| 142 | GE264N | Germany | Northern Germany | SchHols | Hillgroven | 227 | 33 | F8 |
| 143 | GE265N | Germany | Northern Germany | SchHols | Hillgroven | 101 | 7 | D13 |
| 144 | GE266N | Germany | Northern Germany | SchHols | Hillgroven | 256 | 32 | E4 |
| 145 | GE267N | Germany | Northern Germany | SchHols | Hillgroven | 258 | 33 | F8 |
| 146 | GE268N | Germany | Northern Germany | SchHols | Hillgroven | 215 | 32 | E4 |
| 147 | GE271S | Germany | Southern Germany | Bavaria | Wassertrudingen | 270 | 32 | E4 |
| 148 | GE272S | Germany | Southern Germany | Bavaria | Wassertrudingen | 14 | 32 | E4 |
| 149 | GE273S | Germany | Southern Germany | Bavaria | Wassertrudingen | 51 | 33 | E5 |
| 150 | GE274S | Germany | Southern Germany | Bavaria | Wassertrudingen | 179 | 33 | F8 |
| 151 | GE277S | Germany | Southern Germany | Bavaria | Wassertrudingen | 241 | 33 | F8 |
| 152 | GE278S | Germany | Southern Germany | Bavaria | Wassertrudingen | 241 | 33 | F8 |
| 153 | GE279N | Germany | Southern Germany | Bavaria | Wassertrudingen | 172 | 33 | F8 |
| 154 | GE282N | Germany | Northern Germany | LSaxony | Wehnen | 171 | 12 | H6 |
| 155 | GE283N | Germany | Northern Germany | LSaxony | Wehnen | 171 | 12 | H6 |
| 156 | GE284N | Germany | Northern Germany | LSaxony | Wehnen | 209 | 32 | E4 |
| 157 | GE285N | Germany | Northern Germany | LSaxony | Wehnen | 89 | 32 | E4 |
| 158 | GE286N | Germany | Northern Germany | LSaxony | Wehnen | 91 | 16 | F6 |
| 159 | GE287N | Germany | Northern Germany | LSaxony | Wehnen | 218 | 32 | E4 |
| 160 | GE288N | Germany | Northern Germany | LSaxony | Wehnen | 170 | 33 | F8 |
| 161 | GE289N | Germany | Northern Germany | LSaxony | Wehnen | 228 | 23 | I2 |
| 162 | GE290N | Germany | Northern Germany | LSaxony | Wehnen | 231 | 12 | H6 |
| 163 | GE292S | Germany | Southern Germany | Bavaria | Grucking | 29 | 12 | H6 |
| 164 | GE293S | Germany | Southern Germany | Bavaria | Grucking | 29 | 12 | H6 |
| 165 | GE294S | Germany | Southern Germany | Bavaria | Grucking | 57 | 32 | E4 |
| 166 | GE295S | Germany | Southern Germany | Bavaria | Grucking | 57 | 32 | E4 |
| 167 | GE297S | Germany | Southern Germany | Bavaria | Grucking | 29 | 12 | H6 |
| 168 | GE298S | Germany | Southern Germany | Bavaria | Grucking | 57 | 32 | E4 |
| 169 | GE300S | Germany | Southern Germany | Bavaria | Grucking | 57 | 32 | E4 |
| 170 | GE302N | Germany | Northern Germany | SchHols | Bredstedt | 26 | 5 | 1 |
| 171 | GE303N | Germany | Northern Germany | SchHols | Bredstedt | 182 | 8 | D13 |
| 172 | GE304N | Germany | Northern Germany | SchHols | Bredstedt | 182 | 8 | D13 |
| 173 | GE305N | Germany | Northern Germany | Niedersachsen | Burgstemmen | 181 | 26 | 2 |
| 174 | GE306N | Germany | Northern Germany | Niedersachsen | Burgstemmen | 87 | 18 | H4 |
| 175 | GE308N | Germany | Northern Germany | Niedersachsen | Burgstemmen | 232 | 20 | C8 |
| 176 | GE310N | Germany | Northern Germany | Niedersachsen | Burgstemmen | 208 | 33 | F8 |
| 177 | GE315N | Germany | Northern Germany | Niedersachsen | Barnstorf | 21 | 13 | F4 |
| 178 | IRBL682 | Ireland | Ireland | n.a. | n.a. | 269 | 12 | H6 |
| 179 | IRBL683 | Ireland | Ireland | n.a. | n.a. | 64 | 24 | H7 |
| 180 | IRBL684 | Ireland | Ireland | n.a. | n.a. | 27 | 29 | 2 |
| 181 | IRBL685 | Ireland | Ireland | n.a. | n.a. | 83 | 16 | F6 |
| 182 | IRBL686 | Ireland | Ireland | n.a. | n.a. | 100 | 33 | F8 |
| 183 | IRBL689 | Ireland | Ireland | n.a. | n.a. | 35 | 29 | 2 |
| 184 | IRBL690 | Ireland | Ireland | n.a. | n.a. | 139 | 17 | I1 |
| 185 | IRBL691 | Ireland | Ireland | n.a. | n.a. | 253 | 13 | F4 |
| 186 | IRBL692 | Ireland | Ireland | n.a. | n.a. | 226 | 24 | H7 |
| 187 | IRBL693 | Ireland | Ireland | n.a. | n.a. | 226 | 24 | H7 |
| 188 | IRBL694 | Ireland | Ireland | n.a. | n.a. | 48 | 32 | E4 |
| 189 | IRBL695 | Ireland | Ireland | n.a. | n.a. | 226 | 24 | H7 |
| 190 | IRBL697 | Ireland | Ireland | n.a. | n.a. | 39 | 18 | H4 |
| 191 | IRBL698 | Ireland | Ireland | n.a. | n.a. | 272 | 12 | H6 |
| 192 | IRBL703 | Ireland | Ireland | n.a. | n.a. | 134 | 12 | H6 |
| 193 | IRBL705 | Ireland | Ireland | n.a. | n.a. | 73 | 18 | H4 |
| 194 | IRBL706 | Ireland | Ireland | n.a. | n.a. | 248 | 32 | E4 |
| 195 | IRBL709 | Ireland | Ireland | n.a. | n.a. | 239 | 10 | G1 |
| 196 | IRBL715 | Ireland | Ireland | n.a. | n.a. | 257 | 22 | G7 |
| 197 | IRBL720 | Ireland | Ireland | n.a. | n.a. | 216 | 34 | F8 |
| 198 | IRBL721 | Ireland | Ireland | n.a. | n.a. | 221 | 34 | E3 |
| 199 | IRBL723 | Ireland | Ireland | n.a. | n.a. | 31 | 12 | H6 |
| 200 | IRBL724 | Ireland | Ireland | n.a. | n.a. | 222 | 34 | E5 |
| 201 | IRBL726 | Ireland | Ireland | n.a. | n.a. | 19 | 32 | E4 |
| 202 | IRBL728 | Ireland | Ireland | n.a. | n.a. | 211 | 34 | E5 |
| 203 | IRBL729 | Ireland | Ireland | n.a. | n.a. | 166 | 27 | I2 |
| 204 | LI2 | Lithuania | Lithuania | n.a. | n.a. | 103 | 9 | G1 |
| 205 | LI4 | Lithuania | Lithuania | n.a. | n.a. | 103 | 9 | G1 |
| 206 | LI5 | Lithuania | Lithuania | n.a. | n.a. | 247 | 33 | 2 |
| 207 | LI11 | Lithuania | Lithuania | n.a. | n.a. | 245 | 11 | G1 |
| 208 | LI12 | Lithuania | Lithuania | n.a. | n.a. | 3 | 20 | C8 |
| 209 | LI13 | Lithuania | Lithuania | n.a. | n.a. | 249 | 33 | F8 |
| 210 | LI14 | Lithuania | Lithuania | n.a. | n.a. | 245 | 11 | G1 |
| 211 | LI15 | Lithuania | Lithuania | n.a. | n.a. | 245 | 11 | G1 |
| 212 | LI16 | Lithuania | Lithuania | n.a. | n.a. | 245 | 11 | G1 |
| 213 | LI17 | Lithuania | Lithuania | n.a. | n.a. | 3 | 20 | C8 |
| 214 | LI18 | Lithuania | Lithuania | n.a. | n.a. | 249 | 33 | F8 |
| 215 | LI19 | Lithuania | Lithuania | n.a. | n.a. | 198 | 11 | G1 |
| 216 | LI20 | Lithuania | Lithuania | n.a. | n.a. | 133 | 20 | C8 |
| 217 | LI21 | Lithuania | Lithuania | n.a. | n.a. | 34 | 11 | G1 |
| 218 | LI22 | Lithuania | Lithuania | n.a. | n.a. | 34 | 11 | G1 |
| 219 | LI23 | Lithuania | Lithuania | n.a. | n.a. | 34 | 11 | G1 |
| 220 | LI24 | Lithuania | Lithuania | n.a. | n.a. | 79 | 8 | D13 |
| 221 | LI25 | Lithuania | Lithuania | n.a. | n.a. | 6 | 18 | 3 |
| 222 | LI26 | Lithuania | Lithuania | n.a. | n.a. | 243 | 12 | H6 |
| 223 | LI28 | Lithuania | Lithuania | n.a. | n.a. | 243 | 12 | H6 |
| 224 | LI29 | Lithuania | Lithuania | n.a. | n.a. | 243 | 12 | H6 |
| 225 | LI30 | Lithuania | Lithuania | n.a. | n.a. | 243 | 12 | H6 |
| 226 | LI31 | Lithuania | Lithuania | n.a. | n.a. | 6 | 18 | 3 |
| 227 | LI32 | Lithuania | Lithuania | n.a. | n.a. | 79 | 8 | D13 |
| 228 | LI33 | Lithuania | Lithuania | n.a. | n.a. | 79 | 8 | D13 |
| 229 | LI34 | Lithuania | Lithuania | n.a. | n.a. | 59 | 32 | E4 |
| 230 | LI35 | Lithuania | Lithuania | n.a. | n.a. | 58 | 32 | E4 |
| 231 | LI38 | Lithuania | Lithuania | n.a. | n.a. | 244 | 12 | H6 |
| 232 | LI39 | Lithuania | Lithuania | n.a. | n.a. | 244 | 12 | H6 |
| 233 | LI40 | Lithuania | Lithuania | n.a. | n.a. | 244 | 12 | H6 |
| 234 | NO40 | Norway | Norway | n.a. | n.a. | 7 | 32 | E4 |
| 235 | NO41 | Norway | Norway | n.a. | n.a. | 199 | 32 | E4 |
| 236 | NO42 | Norway | Norway | n.a. | n.a. | 200 | 32 | E4 |
| 237 | NO43 | Norway | Norway | n.a. | n.a. | 267 | 32 | E4 |
| 238 | NO44 | Norway | Norway | n.a. | n.a. | 125 | 32 | E4 |
| 239 | NO45 | Norway | Norway | n.a. | n.a. | 240 | 33 | F8 |
| 240 | NO46 | Norway | Norway | n.a. | n.a. | 78 | 32 | E4 |
| 241 | NO47 | Norway | Norway | n.a. | n.a. | 4 | 32 | E4 |
| 242 | NO48 | Norway | Norway | n.a. | n.a. | 178 | 32 | E4 |
| 243 | NO49 | Norway | Norway | n.a. | n.a. | 274 | 32 | E4 |
| 244 | NO50 | Norway | Norway | n.a. | n.a. | 131 | 32 | E4 |
| 245 | NO51 | Norway | Norway | n.a. | n.a. | 188 | 32 | E4 |
| 246 | NO53 | Norway | Norway | n.a. | n.a. | 188 | 32 | E4 |
| 247 | NO55 | Norway | Norway | n.a. | n.a. | 49 | 32 | E4 |
| 248 | NO56 | Norway | Norway | n.a. | n.a. | 271 | 33 | F8 |
| 249 | NO57 | Norway | Norway | n.a. | n.a. | 68 | 8 | D13 |
| 250 | NO59 | Norway | Norway | n.a. | n.a. | 132 | 32 | E4 |
| 251 | NO61 | Norway | Norway | n.a. | n.a. | 24 | 33 | F8 |
| 252 | NO62 | Norway | Norway | n.a. | n.a. | 273 | 32 | E4 |
| 253 | NO63 | Norway | Norway | n.a. | n.a. | 225 | 33 | F8 |
| 254 | NO64 | Norway | Norway | n.a. | n.a. | 46 | 32 | E4 |
| 255 | NO65 | Norway | Norway | n.a. | n.a. | 74 | 33 | F8 |
| 256 | NO66 | Norway | Norway | n.a. | n.a. | 201 | 32 | E4 |
| 257 | NO202652 | Norway | Norway | n.a. | n.a. | 189 | 32 | E4 |
| 258 | SL1 | Slovenia | Slovenia | UppCarniola | Mengei | 214 | 11 | G1 |
| 259 | SL2 | Slovenia | Slovenia | LowCarniola | Sentjakob | 55 | 6 | WILDTYPE |
| 260 | SL3 | Slovenia | Slovenia | UppCarniola | Bitnje | 169 | 11 | G1 |
| 261 | SL5 | Slovenia | Slovenia | LowCarniola | Novomesto | 54 | 6 | WILDTYPE |
| 262 | SL6 | Slovenia | Slovenia | Littoral | Ajdovscina | 217 | 11 | G1 |
| 263 | SL10 | Slovenia | Slovenia | Prekmurje | Beltinci | 52 | 11 | G1 |
| 264 | SW200 | Sweden | Sweden | VastGot | Skovde | 37 | 20 | C8 |
| 265 | SW201 | Sweden | Sweden | VastGot | Skovde | 150 | 14 | F5 |
| 266 | SW202 | Sweden | Sweden | VastGot | Skovde | 8 | 20 | C8 |
| 267 | SW203 | Sweden | Sweden | VastGot | Skovde | 161 | 19 | C4 |
| 268 | SW204 | Sweden | Sweden | VastGot | Skovde | 157 | 20 | C8 |
| 269 | SW205 | Sweden | Sweden | VastGot | Skovde | 155 | 20 | C8 |
| 270 | SW206 | Sweden | Sweden | VastGot | Skovde | 136 | 32 | E4 |
| 271 | SW208 | Sweden | Sweden | VastGot | Skovde | 137 | 30 | C7 |
| 272 | SW209 | Sweden | Sweden | VastGot | Skovde | 148 | 20 | C8 |
| 273 | SW210 | Sweden | Sweden | VastGot | Skovde | 38 | 20 | C8 |
| 274 | SW211 | Sweden | Sweden | VastGot | Skovde | 42 | 20 | C8 |
| 275 | SW212 | Sweden | Sweden | VastGot | Skovde | 147 | 20 | C8 |
| 276 | SW213 | Sweden | Sweden | VastGot | Skovde | 114 | 32 | E4 |
| 277 | SW214 | Sweden | Sweden | VastGot | Skovde | 116 | 20 | C8 |
| 278 | SW215 | Sweden | Sweden | VastGot | Skovde | 159 | 19 | C4 |
| 279 | SW216 | Sweden | Sweden | VastGot | Skovde | 13 | 20 | C8 |
| 280 | SW217 | Sweden | Sweden | VastGot | Skovde | 143 | 11 | G1 |
| 281 | SW218 | Sweden | Sweden | VastGot | Skovde | 152 | 19 | C4 |
| 282 | SW219 | Sweden | Sweden | Ostergot | Motala | 163 | 9 | G1 |
| 283 | SW220 | Sweden | Sweden | Ostergot | Motala | 36 | 11 | G1 |
| 284 | SW221 | Sweden | Sweden | Ostergot | Motala | 154 | 20 | C8 |
| 285 | SW222 | Sweden | Sweden | Ostergot | Motala | 151 | 4 | F8 |
| 286 | SW223 | Sweden | Sweden | Ostergot | Motala | 115 | 20 | C8 |
| 287 | SW225 | Sweden | Sweden | Ostergot | Motala | 10 | 2 | C8 |
| 288 | SW226 | Sweden | Sweden | Ostergot | Motala | 145 | 20 | C8 |
| 289 | SW227 | Sweden | Sweden | Ostergot | Motala | 149 | 32 | E4 |
| 290 | SW228 | Sweden | Sweden | Ostergot | Motala | 152 | 32 | E4 |
| 291 | SW230 | Sweden | Sweden | Ostergot | Motala | 44 | 32 | E4 |
| 292 | SW231 | Sweden | Sweden | Ostergot | Motala | 164 | 20 | C8 |
| 293 | SW232 | Sweden | Sweden | Ostergot | Motala | 16 | 11 | G1 |
| 294 | SW233 | Sweden | Sweden | Ostergot | Motala | 160 | 32 | E4 |
| 295 | SW234 | Sweden | Sweden | Ostergot | Motala | 9 | 20 | C8 |
| 296 | SW235 | Sweden | Sweden | Ostergot | Motala | 138 | 20 | C8 |
| 297 | SW236 | Sweden | Sweden | Ostergot | Motala | 146 | 11 | G1 |
| 298 | SW237 | Sweden | Sweden | Ostergot | Motala | 158 | 20 | C8 |
| 299 | SW238 | Sweden | Sweden | Ostergot | Motala | 76 | 32 | E4 |
| 300 | SW239 | Sweden | Sweden | Skane | Simrishamn | 142 | 20 | C8 |
| 301 | SW241 | Sweden | Sweden | Skane | Simrishamn | 122 | 11 | G1 |
| 302 | SW242 | Sweden | Sweden | Skane | Simrishamn | 43 | 32 | E4 |
| 303 | SW245 | Sweden | Sweden | Skane | Simrishamn | 162 | 33 | F8 |
| 304 | SW248 | Sweden | Sweden | Skane | Simrishamn | 144 | 32 | E4 |
| 305 | SW249 | Sweden | Sweden | Skane | Simrishamn | 106 | 32 | E4 |
| 306 | SW252 | Sweden | Sweden | Skane | Simrishamn | 75 | 32 | E4 |
| 307 | SW253 | Sweden | Sweden | Skane | Simrishamn | 119 | 33 | F8 |
| 308 | SW254 | Sweden | Sweden | Skane | Simrishamn | 45 | 34 | E5 |
| 309 | SW255 | Sweden | Sweden | Skane | Simrishamn | 110 | 33 | F8 |
| 310 | SW256 | Sweden | Sweden | Skane | Simrishamn | 77 | 33 | F8 |
| 311 | SW258 | Sweden | Sweden | Skane | Simrishamn | 156 | 32 | E4 |

Table S2. Reference sequences of the nine housekeeping genes and CYP51 gene and their NCBI reference ID.

Table S3. Characteristics of the SNP loci of the housekeeping gene markers used in this study.

| **Locus** | **Number of alleles** | **Evenness** | **Missing data (%)** |
| --- | --- | --- | --- |
| ACT-21 | 2 | 0.565 | 0.0 |
| ACT-38 | 2 | 0.851 | 0.0 |
| ACT-56 | 1 | n.a. | 0.0 |
| ACT-77 | 2 | 0.652 | 0.0 |
| ACT-93 | 2 | 0.662 | 0.0 |
| ACT-115 | 3 | 0.331 | 0.0 |
| ACT-122 | 2 | 0.657 | 0.0 |
| ACT-137 | 3 | 0.554 | 0.0 |
| ACT-149 | 2 | 0.747 | 0.0 |
| ACT-153 | 2 | 0.733 | 0.0 |
| ACT-156 | 2 | 0.443 | 0.322 |
| ACT-159 | 2 | 0.667 | 0.0 |
| ACT-236 | 2 | 0.628 | 0.0 |
| ACT-252 | 2 | 0.459 | 0.0 |
| ACT-269 | 2 | 0.467 | 0.0 |
| ACT-281 | 2 | 0.425 | 0.0 |
| ACT-296 | 2 | 0.467 | 0.0 |
| ACT-324 | 2 | 0.576 | 0.0 |
| ACT-326 | 2 | 0.459 | 0.0 |
| ACT-341 | 2 | 0.834 | 0.0 |
| ACT-420 | 2 | 0.826 | 0.0 |
| ACT-432 | 2 | 0.416 | 0.0 |
| ACT-503 | 2 | 0.988 | 0.322 |
| ACT-506 | 2 | 0.474 | 0.0 |
| ACT-538 | 2 | 0.994 | 0.0 |
| BTUB-25 | 2 | 0.393 | 0.0 |
| BTUB-28 | 2 | 0.859 | 0.0 |
| BTUB-58 | 2 | 0.874 | 0.0 |
| BTUB-65 | 2 | 0.897 | 0.0 |
| BTUB-70 | 2 | 0.381 | 0.0 |
| BTUB-79 | 3 | 0.818 | 0.0 |
| BTUB-84 | 2 | 0.874 | 0.0 |
| BTUB-99 | 2 | 0.366 | 0.0 |
| BTUB-101 | 2 | 0.874 | 0.0 |
| BTUB-104 | 3 | 0.821 | 0.0 |
| BTUB-112 | 2 | 0.366 | 0.0 |
| BTUB-113 | 2 | 0.889 | 0.0 |
| BTUB-181 | 2 | 0.921 | 0.0 |
| BTUB-199 | 2 | 0.914 | 0.0 |
| BTUB-249 | 2 | 0.945 | 0.0 |
| BTUB-270 | 2 | 0.425 | 0.0 |
| BTUB-279 | 2 | 0.381 | 0.0 |
| BTUB-332 | 2 | 0.481 | 0.0 |
| BTUB-333 | 2 | 0.958 | 0.0 |
| BTUB-387 | 2 | 0.924 | 0.0 |
| BTUB-388 | 2 | 0.955 | 0.0 |
| BTUB-513 | 3 | 0.919 | 0.0 |
| EF1-28 | 2 | 0.787 | 0.0 |
| EF1-32 | 2 | 0.349 | 0.0 |
| EF1-40 | 2 | 0.760 | 0.0 |
| EF1-46 | 2 | 0.349 | 0.0 |
| EF1-69 | 2 | 0.747 | 0.0 |
| EF1-296 | 2 | 0.826 | 0.0 |
| EF1-318 | 2 | 0.905 | 0.322 |
| EF1-398 | 2 | 0.366 | 0.0 |
| EF1-426 | 2 | 0.435 | 0.0 |
| EF1-429 | 1 | n.a. | 0.0 |
| EF1-445 | 2 | 0.886 | 0.0 |
| EF1-455 | 2 | 0.900 | 0.0 |
| EF1-466 | 2 | 0.366 | 0.0 |
| EF1-478 | 2 | 0.893 | 0.0 |
| EF1-498 | 1 | n.a. | 0.0 |
| EF1-526 | 3 | 0.884 | 0.0 |
| GAPDH-59 | 2 | 0.851 | 0.0 |
| GAPDH-83 | 2 | 0.613 | 0.0 |
| GAPDH-125 | 2 | 0.623 | 0.0 |
| GAPDH-140 | 2 | 0.623 | 0.0 |
| GAPDH-164 | 2 | 0.714 | 0.0 |
| GAPDH-203 | 2 | 0.643 | 0.0 |
| GAPDH-245 | 2 | 0.538 | 0.0 |
| GAPDH-301-26 | 3 | 0.712 | 0.0 |
| GAPDH-301-28 | 3 | 0.722 | 0.0 |
| GAPDH-301-31 | 3 | 0.727 | 0.0 |
| GAPDH-311 | 2 | 0.474 | 0.0 |
| GAPDH-386 | 2 | 0.672 | 0.0 |
| GAPDH-422 | 2 | 0.633 | 0.0 |
| GAPDH-449 | 2 | 0.633 | 0.0 |
| GAPDH-488 | 2 | 0.652 | 0.0 |
| GAPDH-494 | 2 | 0.425 | 0.0 |
| GAPDH-508 | 2 | 0.555 | 0.0 |
| GAPDH-533 | 3 | 0.512 | 0.0 |
| Heat-25 | 1 | n.a. | 3.537 |
| Heat-61 | 3 | 0.718 | 3.537 |
| Heat-529 | 2 | 0.615 | 3.537 |
| CYCLO-25 | 2 | 0.381 | 0.0 |
| CYCLO-76 | 2 | 0.501 | 0.0 |
| CYCLO-97 | 2 | 0.501 | 0.0 |
| CYCLO-160 | 2 | 0.501 | 0.0 |
| CYCLO-194 | 2 | 0.990 | 0.0 |
| CYCLO-196 | 2 | 0.481 | 0.0 |
| CYCLO-220 | 2 | 0.501 | 0.0 |
| CYCLO-253 | 2 | 0.623 | 0.0 |
| CYCLO-295 | 2 | 0.393 | 0.0 |
| CYCLO-386 | 2 | 0.405 | 0.0 |
| CYCLO-610 | 2 | 0.826 | 0.0 |
| CALM-123 | 2 | 0.495 | 0.0 |
| CALM-141 | 2 | 0.495 | 0.0 |
| CALM-275 | 2 | 0.532 | 0.0 |
| CALM-356 | 3 | 0.511 | 0.0 |
| CALM-398 | 2 | 0.520 | 0.0 |
| CALM-446 | 1 | n.a. | 0.0 |
| CALM-541 | 2 | 0.481 | 0.0 |
| PKC-246 | 2 | 0.474 | 0.0 |
| PKC-279 | 2 | 0.474 | 0.0 |
| PKC-396 | 2 | 0.733 | 0.0 |
| PKC-4063 | 2 | 0.488 | 0.0 |
| PKC-438 | 2 | 0.366 | 0.0 |
| PKC-636 | 2 | 0.435 | 0.0 |
| TFC1-134 | 2 | 0.999 | 0.0 |
| TFC1-240 | 2 | 0.999 | 0.0 |
| TFC1-336 | 2 | 1.000 | 0.0 |
| TFC1-395 | 2 | 0.451 | 0.0 |
| TFC1-479 | 2 | 0.451 | 0.0 |
|  | **Total: 233** | **Mean: 0.641** | **n.a.** |

Table S4. Characteristics of the SNP loci of CYP51 gene used in this study.

| **Locus** | **Number of alleles** | **Evenness** | **Missing data (%)** |
| --- | --- | --- | --- |
| 1_30 | 2 | 0.366 | 0.0 |
| 1_149 | 2 | 0.459 | 0.0 |
| 1_204 | 2 | 0.459 | 0.0 |
| 1_320 | 2 | 0.327 | 0.0 |
| 1_375 | 2 | 0.796 | 0.0 |
| 1_407 | 3 | 0.765 | 0.0 |
| 2_27 | 2 | 0.805 | 0.0 |
| 2_75 | 2 | 0.805 | 0.0 |
| 2_96 | 2 | 0.805 | 0.0 |
| 2_116 | 2 | 0.805 | 0.0 |
| 2_175 | 2 | 0.366 | 0.0 |
| 2_291 | 2 | 0.843 | 0.0 |
| 2_384 | 2 | 0.847 | 0.0 |
| 4_123 | 2 | 0.822 | 0.0 |
| 4_283 | 2 | 0.443 | 0.0 |
| 4_370 | 2 | 0.327 | 0.0 |
| 4_517 | 2 | 0.349 | 0.0 |
| 4_681 | 2 | 0.618 | 0.0 |
| 4_713 | 2 | 0.967 | 0.0 |
| 4_738 | 2 | 0.984 | 0.0 |
| 4_768 | 2 | 0.984 | 0.0 |
|  | **Total 29** | **Mean 0.664** | **n.a.** |


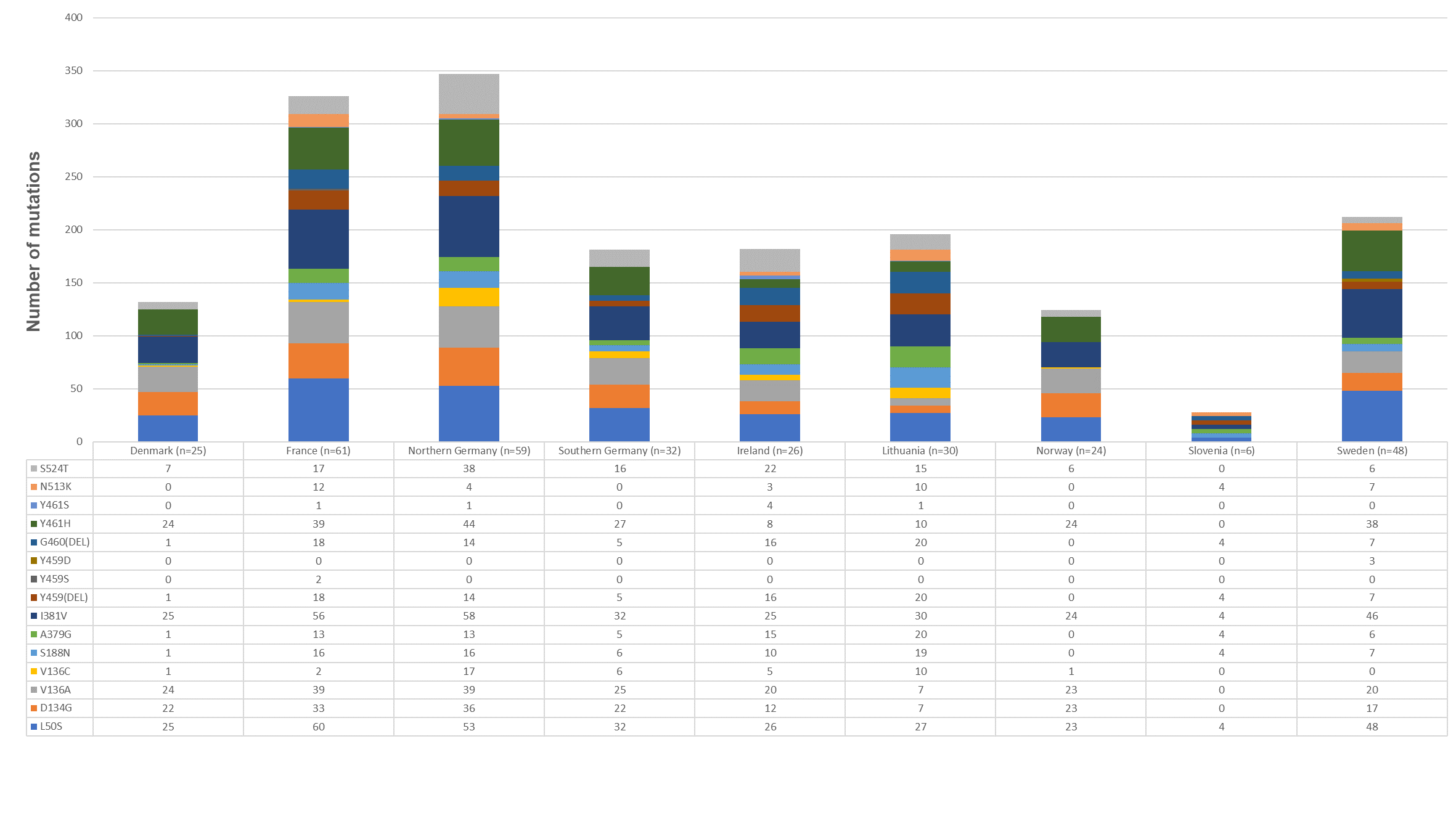
Figure S1. Distibution of 15 mutations in the CYP51 gene in *Zymoseptoria tritici* populations across Europe in the year 2019.


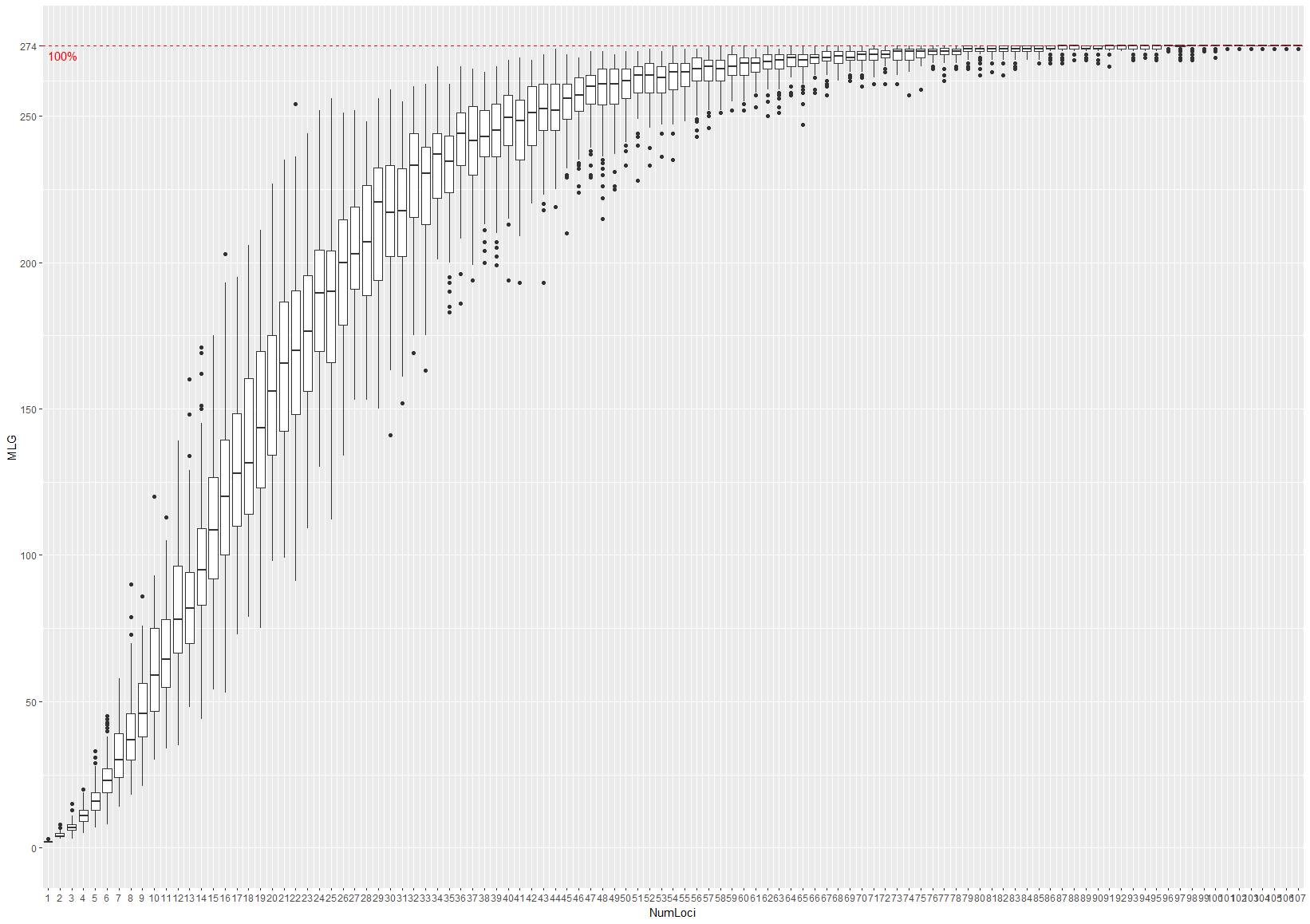


Figure S2. Genotype accumulation curve of 311 *Zymoseptoria tritici* isolates genotyped based on housekeeping genes obtained with poppr package. The red dashed line represents 100% of the total observed genotypes.


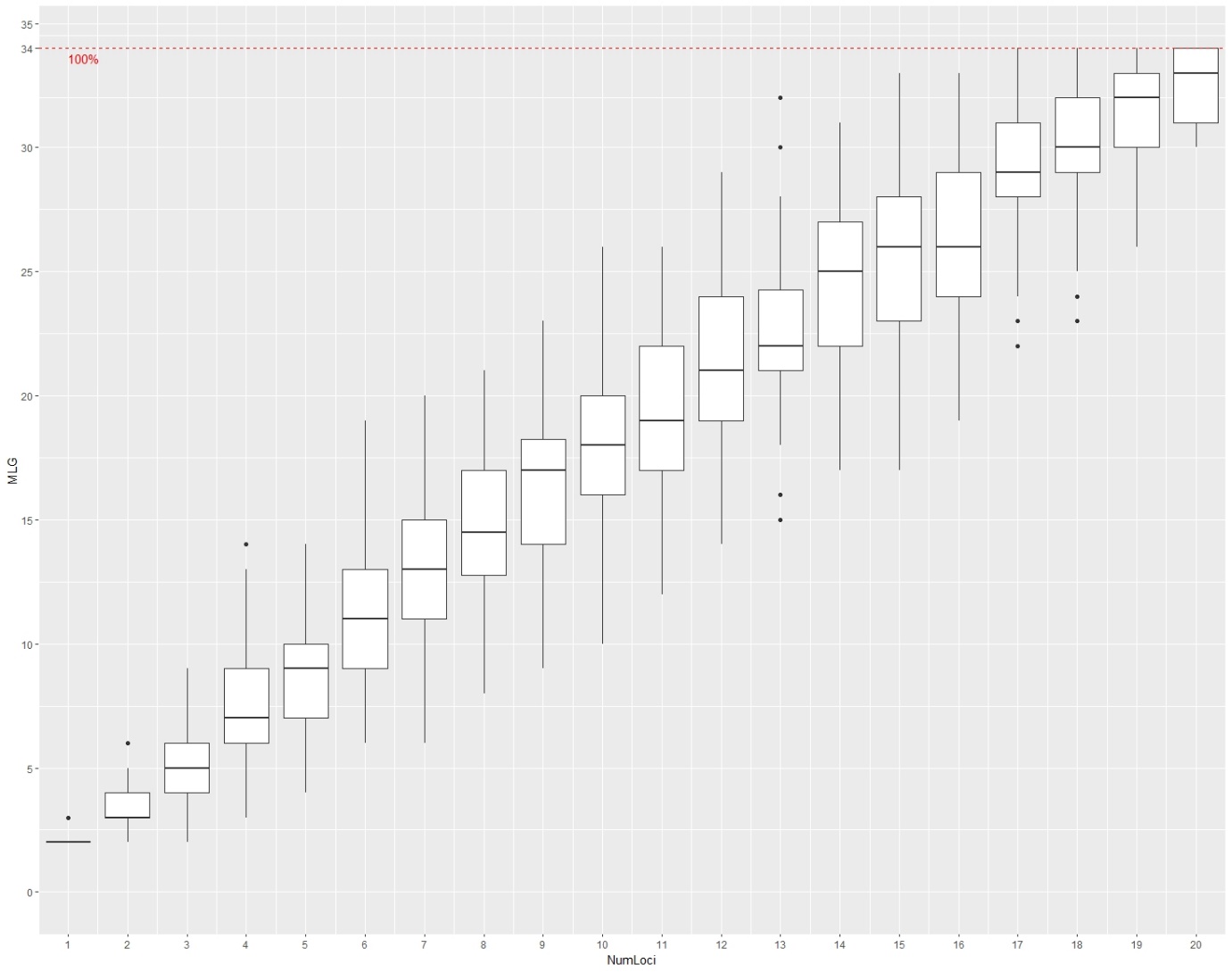


Figure S3. Genotype accumulation curve of 311 *Zymoseptoria tritici* isolates genotyped based on CYP51 gene obtained with poppr package. The red dashed line represents 100% of the total observed genotypes.


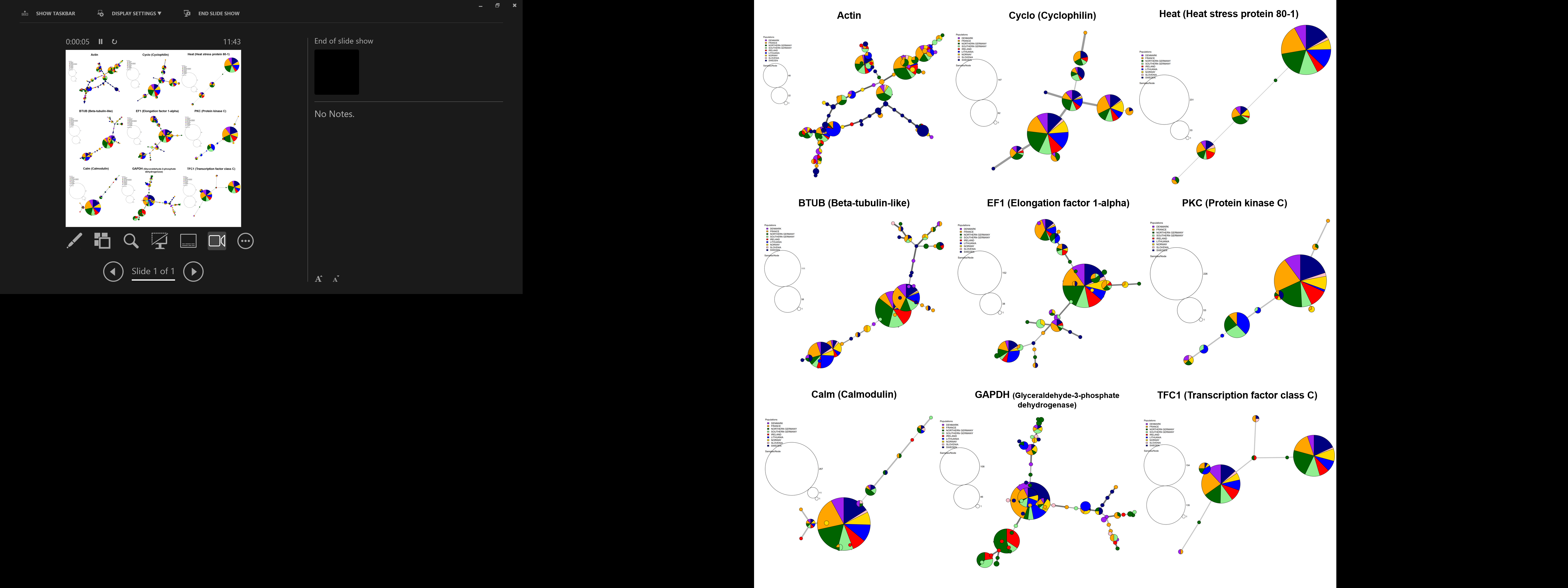


Figure S4. Minimum spanning network (MSN) of *Zymoseptoria tritici* based on Provesti’s distance analyzed in each housekeeping gene used in this study. Each circle represents a unique multilocus genotype (MLG), branch thickness represents genetic relatedness, and MLGs shared between regions are indicated by split nodes.
